# Comparison of clinical effects of two surgical approaches in the treatment of acetabular fractures

**DOI:** 10.12669/pjms.39.4.6914

**Published:** 2023

**Authors:** Bing Liu, Hongtao Shang, Guixian Dong, Ning Zhang, Shengjie Wang

**Affiliations:** 1 Bing Liu, Department of Traumatic Orthopedics, Hengshui City People’s Hospital, Harrison International Peace Hospital, Hengshui 053000, Hebei, China; 2 Hongtao Shang, Department of Traumatic Orthopedics, Hengshui City People’s Hospital, Harrison International Peace Hospital, Hengshui 053000, Hebei, China; 3 Guixian Dong, Department of Traumatic Orthopedics, Hengshui City People’s Hospital, Harrison International Peace Hospital, Hengshui 053000, Hebei, China; 4 Ning Zhang, Department of Traumatic Orthopedics, Hengshui City People’s Hospital, Harrison International Peace Hospital, Hengshui 053000, Hebei, China; 5 Shengjie Wang, Department of Traumatic Orthopedics, Hengshui City People’s Hospital, Harrison International Peace Hospital, Hengshui 053000, Hebei, China

**Keywords:** Acetabular fracture, Lateral rectus abdominis approach, Improved Stoppa approach, Clinical effect

## Abstract

**Objective:**

To determine the clinical effect of lateral rectus abdominis approach and modified Stoppa approach for the surgical treatment of acetabular fractures.

**Methods:**

A retrospective analysis was performed on the case data of 30 patients with acetabular fractures admitted to the Department of Orthopaedics of Hengshui City People’s Hospital from June 2017 to June 2021. According to the surgical methods, the enrolled patients were divided into the lateral rectus abdominis approach group (observation group) and the modified Stoppa approach group (control group), with 15 patients in each group. Further comparison was made on the incision length, operation time, intraoperative blood loss, length of stay in the hospital, fracture reduction, hip joint function, neurological recovery, and postoperative complications between the two groups.

**Results:**

There was no significant difference between the two groups in the length of stay in the hospital, hip joint function score, fracture reduction quality, and excellent-to-good rate of hip joint function (*p>*0.05). There were significant differences in incision length, intraoperative blood loss, operation time, postoperative motor and touch function scores, and postoperative complication rate between the observation group and the control group (*p<*0.05).

**Conclusion:**

The clinical effect of the lateral rectus abdominis approach is close to that of the modified Stoppa approach for the surgical treatment of acetabular fracture patients. However, and importantly, surgery through the lateral rectus abdominis approach has less trauma, shorter operation time, lower surgical complications, and good postoperative functional recovery.

## INTRODUCTION

With the rapid development of communications, transportation and construction industries, it triggers a serious problem of the growing number of acetabular fractures caused by high-energy trauma, leading to an annual increase of the disability and mortality rates of the affected patients.^1^ The location of acetabular fracture is generally deep in anatomy, with the distribution of a large number of nerves and blood vessels around, resulting in difficult surgical exposure and reduction.^2^ The majority of patients with acetabular fracture have poor clinical prognosis.^3^ It has been reported that appropriate surgical approach can significantly improve the surgical effect of acetabular fracture and improve the prognosis of patients with fracture.^4^

The classic surgical approach for acetabular fractures is the ilioinguinal approach. Nevertheless, its clinical effect is poor since surgery through this approach may induce large trauma, have complex operation, high technical requirements, postoperative infection, cause inguinal hernia formation and other complications.^5^,^6^ The approaches that are widely used clinically for the surgical treatment of acetabular fractures include the improved Stoppa approach, Kocher-Langenbeck approach, as well as the combined anterior posterior approach. At present, the lateral rectus abdominis approach is extensively applied in clinical. However, it is still a controversial issue concerning the clinical effect of surgery via this approach.^7^ In view of the above, the present study was carried out to compare the clinical effects of the lateral rectus abdominis approach and the modified Stoppa approach for the surgical treatment of acetabular fractures, with the purpose to provide possible basis for determining the surgical program of acetabular fractures.

## METHODS

This is a retrospective study. The subjects of study were 30 patients with acetabular fractures treated in the Department of Orthopaedics of Hengshui City People’s Hospital from June 2017 to June 2021. According to the random number table method, the enrolled patients were divided into the lateral rectus abdominis approach group (observation group) and the modified Stoppa approach group (control group), with 15 patients in each group. After admission, all patients received external fixation with pelvic external fixator or lower-limb skeletal traction according to the patients’ condition before operation. The patients and their families were informed of the choice of surgery and provided written informed consent for surgery.

### Ethical Approval:

The study was approved by the Institutional Ethics Committee of Hengshui City People’s Hospital (No.: 2022-2-011; Date: June 30, 2022), and written informed consent was obtained from all participants.

### Inclusion criteria:

- Patients who aged >18 years old with fresh fractures;
- Patients without symptoms of vascular and nerve injury before operation;
- Patients who had no wound infection before operation and whose fracture conditions allowed internal fixation;
- Patients with complete clinical data.

### Exclusion criteria:

- Patients with mental disorder and cognitive impairment;
- Patients with with severe heart, liver, kidney and other important organ diseases;
- Patients who could not follow the doctor’s advice for rehabilitation treatment after operation;
- Patients with diabetes, osteoporosis and malignant tumors.
- Pregnant or lactating women;

Patients in the observation group received surgery through the lateral rectus abdominis approach. An incision was made on the line between the two points where the starting point was at the 1/3 of the medial and lateral area of the line between the anterior superior iliac spine and the umbilical cord, and the ending point was the midpoint of the inguinal ligament. The anterior sheath of rectus abdominis was incised at the junction of rectus abdominis and internal oblique abdominis. After the rectus abdominis was fully exposed, blunt separation was performed along the lateral rectus muscle to the extraperitoneal space, so as to fully expose the 1^st^, 2^nd^, and 3^rd^ windows. Acetabular fracture was reduced under direct vision. After confirming the good reduction of the fracture, the fracture was fixed after shaping using reconstructive titanium plate.

Patients in the control group were provided surgery via the modified Stoppa approach. A longitudinal incision about 12 cm in length was made in the middle of the lower abdomen of the line from pubic symphysis to umbilicus. The Hunter’s line was cut longitudinally from bottom to top to enter into the outside of the extraperitoneal area. The peri iliac fascia was dissected to expose the fracture end. Fracture was reduced under direct vision and temporary fixed with Kirschner wire. After satisfactory reduction confirmed by C-arm fluoroscopy, the fracture was fixed with reconstructive titanium plate. Patients in both groups underwent regular functional exercise after operation.

The patients were followed up by outpatient reexamination and telephone for 8-18 months postoperatively. Patients in both groups received the same standard of nursing intervention. The perioperative outcome measures of the two groups were analyzed and compared, including operation time, incision length, intraoperative blood loss, hip joint function score and postoperative complications. The quality of fracture reduction of the two groups was determined according to the image criteria of Matta^3^:

- ***Aanatomical reduction:*** The offset distance of fracture reduction was within one mm;
- ***Satisfactory reduction:*** within one-three mm; and

***Unsatisfactory reduction:*** over three mm. The hip joint function of the two groups was scored by the modified Merled’Aubigne-Postel scoring system 6 months after operation, which was classified into 4 grades of excellent (18 points), good (15-17 points), general (12-14 points) and poor (<12 points). In addition, the motor and touch scores of the American Spinal Injury Association (ASIA) were used to evaluate the neurological recovery of the two groups.

### Statistical analysis:

SPSS 21.0 statistical software was used to analyze the statistical differences of data between groups. The measurement data were expressed in `x±s and compared using *t* test between groups; the counting data were described in rate and compared using c² test between groups. There was a statistically significant difference when *p<*0.05.

## RESULTS

As shown in Table-I, there was no significant difference between the two groups in general data before operation (*p>*0.05). No significant difference was observed in the length of stay in the hospital and hip joint function score between the two groups (*p>*0.05). While the observation group showed shorter incision length, less amount of intraoperative blood loss, and shorter operation time than those of the control group (*p<*0.05; Table-II). There was no significant difference in fracture reduction quality between the two groups (*p>*0.05; Table-III). There was no significant difference in the excellent-to-good rate of hip joint function between the two groups (*p>*0.05; Table-IV).

**Table-I:** Comparison of general data between the two groups.

Indexes	Observation group (n=15)	Control group (n=15)	t/χ^2^	P
Age (years)	45.47±10.64	46.60±10.45	0.294	0.771
Male [n (%)]	8 (53.33%)	7 (46.67%)	0.133	0.715
Fracture type			0.556	0.456
Simple	10 (66.67%)	5 (33.33%)		
Complex	8 (53.33%)	7 (46.67%)		
Cause of trauma			0.182	0.913
Traffic injury	5 (33.33%)	6 (40.00%)		
High fall injury	4 (26.67%)	4 (26.67 %)		
Bruise	6 (40.00%)	5 (33.33%)		
Interval from injury to operation	8.13±2.59	8.53±2.56	0.426	0.674

**Table-II:** Comparison of perioperative indexes between the two groups (*χ̅*±*S*).

Groups	Incision length (cm)	Intraoperative blood loss (ml)	Operation time (min)	Length of stay in the hospital (d)	Hip joint function score (points)
Observation group	8.00±0.65	357.00±7.27	155.07±5.16	14.13±2.10	15.87±1.55
Control group	9.47±0.99	388.00±8.82	160.33±6.58	14.20±1.70	16.07±2.12
*t*	4.785	10.501	2.440	0.096	0.295
*P*	0.000	0.000	0.021	0.925	0.770

**Table-III:** Comparison of fracture reduction quality between two groups [n(%)].

Reduction quality	Observation group (n=15)	Control group (n=15)	c^2^	P
Anatomical reduction	7 (46.67)	6 (40.00)	0.136	0.713
Satisfactory reduction	7 (46.67)	8 (53.34)	0.133	0.715
Unsatisfactory reduction	1 (6.66)	1 (6.66)	0.000	1.000

**Table-IV:** Comparison of excellent-to-good rate of hip joint function between the two groups [n(%)].

Groups	Excellent	Good	General	Poor	Excellent-to-good rate
Observation group	6 (40.00)	8 (53.34)	1 (6.66)	0 (0.00)	14 (93.33)
Control group	5 (33.33)	7 (26.67)	2 (20.00)	1 (13.33)	9 (80.00)
c^2^					1.154
*P*					0.283

Preoperative comparison revealed no significant difference in ASIA motor and touch scores between the two groups (*p>*0.05). One year after operation, the motor and touch scores of the observation group were significantly higher than those of the control group (*p<*0.05; Table-V).

**Table-V:** Comparison of ASIA motor and sensory scores between the two groups (points,*χ̅*±*S*).

Groups	ASIA motor score		ASIA sensory score	

	Before operation	1 year after operation	Before operation	1 year after operation
Observation group	34.40±7.40	79.20±6.04	44.13±5.03	75.00±4.31
Control group	37.33±7.58	53.87±6.89	45.00±6.41	56.07±7.98
*t*	1.072	10.714	0.412	8.087
*P*	0.293	0.000	0.684	0.000

The postoperative rate of complications of the observation group was significantly lower than that of the control group (*p<*0.05; Table-VI).

**Table-VI:** Comparison of postoperative complications between the two groups [n(%)].

Groups	Incision infection	Pulmonary infection	Urinary tract infection	Others	Rate of complications
Observation group	1 (6.67)	0 (0.00)	0 (0.00)	1 (6.67)	2 (13.33)
Control group	1 (6.67)	2 (13.33)	3 (20.00)	1 (6.67)	7 (46.67)
*c* ^2^					3.968
*P*					0.046

## DISCUSSION

Acetabular fractures are becoming increasingly more common clinically at present.^8^ In the absence of timely and effective treatment, there may be poor prognosis, and it can lead to the death of patients in serious cases.^9^ Surgical treatment is the preferred option for the treatment of acetabular fractures. While the surgical effect is positively related to the quality of fracture reduction, and the latter is closely related to the surgical approach.^10^,^11^ Posterior approaches are commonly used clinically, among which Kocher-Langenbeck is the most classical method. However, surgery through this approach has a high risk of iatrogenic nerve injury and is technically difficult.^12^ There have been many clinical studies on the anterior approaches, such as the ilioinguinal approach, the modified Stoppa approach and the lateral rectus abdominis approach. However, there are still many disputes concerning the clinical choice of the anterior approaches.^13^,^14^ In the present study, the clinical effect and surgical complications of two new anterior approaches were analyzed in the treatment of acetabular fractures.

According to prior clinical research,^15^ the lateral rectus abdominis approach and the modified Stoppa approach are the main surgical choices for the treatment of acetabular fractures. In our study, there was no statistically significant difference of the length of stay in the hospital, fracture reduction quality, hip joint function score and excellent-to-good rate of hip joint function between the two groups. These results suggest that surgery through the two approaches is both simple in operation and can clearly expose the fracture, have little impact on hip joint function of patients and can lead to high patient satisfaction. It has been reported that there was a significant difference in incision length between the lateral rectus abdominis approach and the modified Stoppa approach.^16^-^18^ Meanwhile, the operation time and intraoperative blood loss of patients with surgery through the lateral rectus abdominis approach were significantly less than those with modified Stoppa approach.^19^ In our study, the observation group showed shorter incision length and less amount of intraoperative blood loss than those of the control group, with statistically significant differences (*p<*0.05). These results support that under the condition of similar clinical effect, surgery through the lateral rectus abdominis approach is less invasive, has smaller intraoperative injury and shorter operation time than the modified Stoppa approach. Notably, the lateral rectus abdominis approach has the following advantages compared with the modified Stoppa approach. To be specific, through this approach, a conventional abdominal exploration incision is made and the operation is simple without involvement of the main nerves and blood vessels; and it is a longitudinal incision with little damage. Besides, it can reduce the fractures of the true pelvic ring and the medial side of the iliac bone under direct vision through this approach; and it can provide convenience for the posterior approach.

Furthermore, according to the comparison of the ASIA motor and touch scores of the two groups one year after operation, the postoperative motor and touch scores of patients with surgery through the lateral rectus abdominis approach were significantly better than those with modified Stoppa approach, indicating that patients undergoing the former surgical procedure had better neurological function recovery. Simultaneously, the rate of postoperative complications in patients with surgery through the lateral rectus abdominis approach was much lower than that via the modified Stoppa approach, suggesting that the former surgical procedure can effectively reduce the risk of postoperative complications in patients with acetabular fractures. Some previous studies^20^ have documented that there was no significant difference in the incidence of postoperative complications for patients treated by surgery through the lateral rectus abdominis approach and the modified Stoppa approach. However, our study revealed some different results, which may be explained by the relatively small sample size, higher proportion of simple fractures and relatively young patients in this study. It may also be related to the short operation time and postoperative use of antibiotics. With respect to the above, findings in our study may provide potential reference that the appropriate surgical approach and timing can be determined according to the condition of patients in clinical practice, so as to improve the clinical surgical effect in treating patients with acetabular fracture.

### Limitations of this study:

It includes small sample size, short period of follow-up. The clinical effects of surgery through the two approaches still remain to be confirmed by further studies with larger sample size and long-term clinical follow-up.

## CONCLUSION

Compared with the modified Stoppa approach, surgery through the lateral rectus abdominis approach has controllable operation time, can fully expose the fracture intraoperatively, without impact by bladder lesions, which is conducive to improving the quality of reduction. Moreover, surgery through this approach has smaller incision, shorter operation time, less intraoperative blood loss, and low risk of postoperative complications.

### Authors’ Contributions:

**BL and HS** designed this study, prepared this manuscript, are responsible and accountable for the accuracy and integrity of the work.

**GD and** **NZ** collected and analyzed clinical data.

**SW: Data analysis,** significantly revised this manuscript.
